# Direct measurement of plume velocity to characterize point source emissions

**DOI:** 10.1073/pnas.2507350122

**Published:** 2025-09-02

**Authors:** Michael L. Eastwood, David R. Thompson, Robert O. Green, Jay E. Fahlen, Taylor J. Adams, Adam R. Brandt, Philip G. Brodrick, Adam Chlus, Eric A. Kort, Frances Reuland, Andrew K. Thorpe

**Affiliations:** ^a^Jet Propulsion Laboratory, California Institute of Technology, Pasadena, CA 91109; ^b^Climate and Space Sciences and Engineering, University of Michigan, Ann Arbor, MI 48109; ^c^Department of Energy Science and Engineering, Standford University, Stanford, CA 94305

**Keywords:** atmospheric methane, remote sensing, imaging spectroscopy

## Abstract

An explosion of recent research uses remote imaging spectroscopy from aircraft and spacecraft to detect and quantify methane point source emissions. These instruments first map the methane enhancement field and then combine this information with the effective wind speed to estimate the source emission rate. This wind speed is typically the largest uncertainty in derived emission rates. It is often, by necessity, inferred from coarse-resolution meteorological reanalysis products which do not match the spatial or temporal extent of wind experienced by the gas plume. Here, we circumvent this problem by simultaneously measuring plume velocity using the same spectrometer that maps the methane plume. Our approach acquires multiple consecutive views of the same point source, with visual tracking of the plume’s features to estimate its ground velocity. This resolves the representational mismatch between reanalysis and effective wind speeds. It provides data with exact spatiotemporal coincidence to the plume being measured. The approach facilitates dramatic improvement in the precision of remote methane point source quantification.

Remote imaging spectrometers are an important emerging tool for monitoring and mitigating facility scale anthropogenic methane emissions. Just a decade has passed since the first large-scale airborne campaigns (1) and orbital detections (2), but spectroscopic detection of methane point sources is already an operational capability and key focus of satellite missions including GHGSat (3), EMIT (4), and CarbonMapper (5). Remote mapping can find unknown emissions, characterize known emitters, and confirm leak remediation for this potent greenhouse gas.

Wind speed at the surface is the biggest source of uncertainty in remote methane point source quantification (6, 7). Remote spectrometers cannot measure emissions directly; instead they measure the absorption of photons by atmospheric methane, which indicates the column density and therefore plume mass. This mass is related to emission rate by the wind speed, e.g. doubling the wind speed carries methane downwind twice as fast, halving the enhancement in a given plume area. Analysts typically use wind speed from reanalysis products (6), but this leaves large uncertainties due to the products’ coarse spatial and temporal resolutions. The effective wind speed experienced by the plume, including effects of vertical spreading and topography, is a different quantity from the wind speed reported in meteorological reanalysis (6). While instruments and algorithms continue advancing, wind speed remains a stubborn uncertainty in remote point source quantification.

Here, we overcome this uncertainty by using the plume itself as a passive tracer, enabling simultaneous measurement of velocity and mass from the same instrument. An optical flow approach acquires multiple views of the source separated by a few seconds and tracks visual features of the methane plume to measure its velocity. Optical velocimetry is an established technology for estimating fluid flow in underwater plumes (8). In open air, similar methods have been used to measure volcanic SO_2_ emissions (9) and ground-level methane leaks (10). However, the approach has not yet been applied to overhead remote methane monitoring. Optical tracking requires multiple views of a plume some seconds apart. In this demonstration, we used changes in aircraft pitch angle to collect time-spaced images. Future instruments could have multiple view angles to enable plume tracking in level flight. We evaluate the approach on an aircraft-mounted sensor sampling a controlled release of methane where emissions are known and wind speed is directly observed at 2 and 10 m. Measuring plume velocity simultaneously with plume detection promises to significantly improve the accuracy of remote methane retrievals.

## Methods

## Materials and Methods

We conducted a field test using the AVIRIS-3 airborne imaging spectrometer, which measures ground-reflected solar photons from 380 to 2,500nm at 7.4nm sampling, with a Signal to Noise Ratio peaking above 1,200 in the methane absorption region for well-illuminated scenes (11). The instrument was mounted onboard a King Air B-200 aircraft which overflew a controlled methane release in Casa Grande, Arizona (38.82N, 111.78W). The ground site conducted a controlled release of methane from a height of 7.3 m above ground level, with methane emissions controlled and quantified with high precision and accuracy using an approach outlined by El Abbadi et al. (12). The site included a sonic anemometer 10 m above ground level and vertically resolved LIDAR wind speed measurements up to 100 m elevation. To account for the time history of the controlled emission rate and wind, we averaged the release rate over the preceding minute. Following protocols in prior work (1, 2, 4), we calibrated the raw sensor data to units of spectral radiance at sensor, and then applied a matched filter to estimate the methane enhancement.

AVIRIS-3 is a pushbroom instrument with a nadir perspective, measuring the terrain under the aircraft with a ±20^°^ cross-track field of view (11). Typically, the sensor flies a level flight path, its field of view building up a long rectangular image. Here, the aircraft flew in a unique multisegment path to obtain multiple views of the target. At the appropriate approach distance from the emission tower, the aircraft transitioned from level flight to a 15^°^ climb to observe the plume ahead of the aircraft position. A real-time methane detection display allowed operators to see the target plume as it passed through the sensor field of view. After the plume was completely imaged, the aircraft quickly transitioned to a pitch-down orientation causing the instrument field of view to swing backward behind the aircraft’s nominal nadir view position. During this transition, the plume was imaged a second time. The aircraft then flew 15^°^ pitch-down for several seconds while AVIRIS-3 acquired a rearward-looking pushbroom image of the target. Upon completing this third plume image the aircraft returned to level flight. This maneuver resulted in three images of the plume with consecutive frames separated by 15 and 12 s. All spectra were geolocated independently using a Global Positioning System and Inertial Measurement Unit mounted to the sensor and a geometric camera model. We traced the view direction of each spectrum until it intersected the local digital elevation model, determining the geographic footprint of that spectrum. This produced a three-frame, 27 s georeferenced movie of the enhancement field with spatial sampling of 2.5 m and a spatial extent of 880 × 1,640 m.

Plume tracking is numerically overdetermined with each image pair providing thousands of pixelwise column density measurements that could be combined in multiple ways to estimate velocity and enhancement. Here, we estimated the plume velocity in each pair of adjacent frames using Lucas/Kanade optical flow (13). This determined the local motion at a selected set of corner points, i.e. locations on the plume with high optical contrast. We filtered features that were either stationary or had low methane values, indicating that they were part of the background. We then calculated the median of local Lucas/Kanade velocity vectors, which was more resilient than a mean to any residual outliers from background clutter. We translated the median optical flow into geographic units to estimate plume velocity. Apart from wind, parallax induced by change in the observer’s location also caused apparent motion of the elevated plume relative to the terrain. The Lukas/Kanade features had an average distance from the source of 1,173 m, for which a classical Briggs plume rise model (14) predicted a height of 41.8 m and a corresponding parallax motion of −0.83 m s^−1^. We subtracted this value from the downtrack component of the plume velocity to compensate. Finally, we calculated the emission rate at source using the Integrated Methane Enhancement (IME) approach (4). We calculated the flux uncertainty due to plume velocity estimation by bootstrap sampling over the tracked Lucas/Kanade features. This accounted for errors such as background interference or ambiguous features that could create spurious velocity vectors. Finally, we compared fluxes derived from image-estimated plume velocities to conventional wind speeds from the HRRR and ERA5 reanalysis products and in situ instrumentation.

## Results

**Fig. 1.**
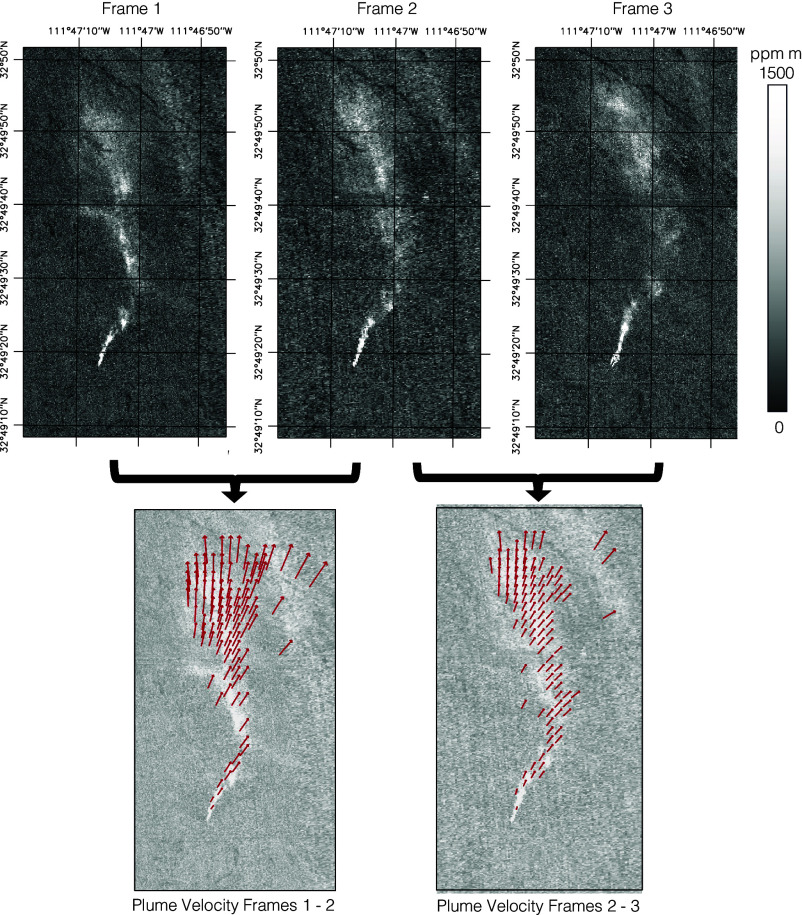
Multi-image sequence of methane plume enhancement images, acquired on a three-segment trajectory. The *Top* row shows the first, second, and third CH4 enhancement fields, with grid lines showing latitude and longitude. The images show methane enhancement in units of ppm m. The *Bottom* row shows derived plume velocities with red arrows indicating optical flow. In both cases, dozens of features are tracked. For visualization, we interpolate this optical flow result using a spatial Gaussian process.

**Fig. 2.**
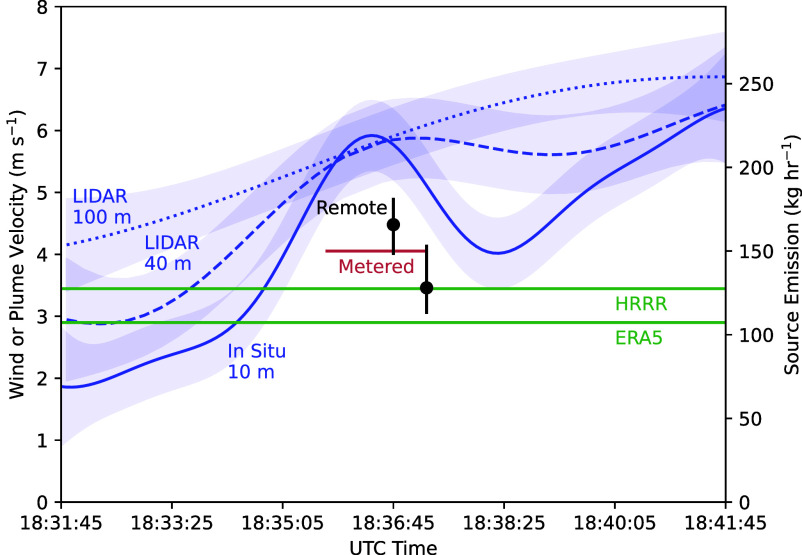
Field wind speed measurements and reanalysis predictions compared to remote plume velocity measurements. We translate between emission rate and the wind speed (plume velocity) using the IME approach of Thorpe et al. (4). Error bars show 95% CIs. Field measurements of wind speed use Gaussian process interpolation with a radial basis function kernel.

Fig. 1 shows the evolving methane enhancement field, with vectors in the Bottom row representing plume velocity between each image pair. We estimated the average plume velocity to be 4.0 m s^−1^, compared to reanalysis wind speed estimates of 3.4 m s^−1^ (HRRR) and 2.9 m s^−1^ (ERA5). The 10 m sonic anemometer measured average wind speed of 5.7 m s^−1^, broadly consistent with LIDAR data from all elevations during the overpass. Fig. 2 compares these values.

The remote plume velocity approach estimated the flux to be 165.4 kg h^−1^ in the first image pair and 128.0 kg h^−1^ in the second image pair. The average estimate of 148.2 kg h^−1^ compared favorably to the true flux metered at approximately 150 kg h^−1^. In contrast, HRRR reanalysis predicted a flux of 127.1 kg h^−1^, ERA5 predicted 107.45 kg h^−1^, and the 10 m anemometer predicted 211.6 kg h^−1^.

## Discussion

The two plume velocity measurements are not statistically independent because they share a common image frame. Nevertheless, we find neither to be inconsistent with the metered flux after accounting for the variability of the feature tracking process. Notably, wind speeds derived from reanalysis data also provide a reasonable flux retrieval. This alignment is likely coincidental, since reanalysis predictions are hourly averages, and the wind speed varies between 2 to 7 m s^−1^ in the ten minutes surrounding the overpass (Fig. 2). Moreover, optical flow indicates the velocity field varies over the hundreds of meters imaged by AVIRIS-3. In contrast, reanalysis models operate at 3 km or coarser. Field measurements at different elevations differ by 50% for some periods. It is precisely this difficulty in defining the “correct” wind speed that motivated our approach.

While we focused here on methane, representational mismatch is a challenge for remote sensing of all gas point sources. Conventional flux estimation approaches require the effective wind speed $U_{\text{eff}}$ experienced by the plume of a given spatial extent, situated in rough topography, which may differ from the local average wind speed which is either measured or inferred from meteorological database. Varon et al. estimate typical wind speed-induced errors as 15% to 50% for IME flux estimation (6). Direct plume velocity measurement overcomes these errors of scale and representation, since the velocity of the plume is definitionally equivalent to $U_{\text{eff}}$. Moreover, the two-dimensional velocity track collapses the vertical dimension, just like the remote column density measurement.

This study is a proof of concept that leaves room for further development. Additional experiments could refine the optical flow approach and quantify its uncertainty. In the long term, new acquisition approaches could obviate the need for atypical aircraft maneuvers. Alternative instrument configurations such as a continuously scanning mirror could enable optical tracking in level flight. From orbit, spacecraft could track plumes by sweeping the field of view multiple times across the target using a scanning mirror or an agile platform. Their coarser ground sampling distance would provide more time to rescan the field. As an extreme example, Watine et al. (15) track plume advection over multiple hours from a geostationary platform. However, orbital ground sampling must be fine enough to resolve trackable features, making lower orbits preferable for all but the largest sources. Since its inception, remote greenhouse point source monitoring has relied on either coarse-grained meteorological products, or rarely, sparse point measurements from in situ anemometers. Plume feature tracking provides a view of the local plume velocity field at the scale most relevant for emissions estimation. More significantly, it may ultimately relieve the assumption of spatially uniform plume velocity, enabling even more sophisticated retrieval algorithms than those of today.

## Data Availability

All PNG image data, field data, python code, and Excel spreadsheets necessary for reproducibility have been deposited in an archive at https://doi.org/10.5281/zenodo.16782430 (16). The remote spectroscopy data used in this paper are archived permanently at: https://doi.org/10.5281/zenodo.15185461. Our matched filter code is available at: https://doi.org/10.5281/zenodo.15127807. Bespoke code and field data for this manuscript will be uploaded to Zenodo upon acceptance (Airborne data DOI: 10.5281/zenodo.15185461 (17), Matched filter DOI: 10.5281/zenodo.15127807 (18). A DOI for the field data and manuscript-specific code will be obtained using the final publication-ready version).
